# Our Defences

**Published:** 1876-07

**Authors:** 


We have occasionally seen exceptions taken to some of our teachings, and no doubt others have occurred which have not met our eye. We wish to inform our readers, once for all, we write for utility. Nothing is radically and permanently useful which is not true; hence it is our effort always to be literally correct in all our statements, and those statements are made either as the result of our own observation or the *corroborated* observations of scientific men recorded in standard publications.

